# Prevalence of retinopathy among adults with self-reported diabetes mellitus: the Sri Lanka diabetes and Cardiovascular Study

**DOI:** 10.1186/1471-2415-14-100

**Published:** 2014-08-20

**Authors:** Prasad Katulanda, Priyanga Ranasinghe, Ranil Jayawardena

**Affiliations:** 1 Diabetes Research Unit, Department of Clinical Medicine, Faculty of Medicine, University of Colombo, Kynsey Road, Colombo, Sri Lanka; 2 Oxford Centre for Diabetes, Endocrinology and Metabolism, University of Oxford, Oxford, UK; 3 Department of Pharmacology, Faculty of Medicine, University of Colombo, Colombo, Sri Lanka; 4 Institute of Health and Biomedical Innovation, Queensland University of Technology, Brisbane, QLD, Australia

**Keywords:** Diabetic retinopathy, Diabetes mellitus, Prevalence, Adults, Sri Lanka

## Abstract

**Background:**

At present there are no large scale nationally-representative studies from Sri Lanka on the prevalence and associations of Diabetic Retinopathy (DR). The present study aims to evaluate the prevalence and risk factors for DR in a community-based nationally-representative sample of adults with self-reported diabetes mellitus from Sri Lanka.

**Methods:**

A cross-sectional community-based national study among 5,000 adults (≥18 years) was conducted in Sri Lanka, using a multi-stage stratified cluster sampling technique. An interviewer-administered questionnaire was used to collect data. Ophthalmological evaluation of patients with ‘known’ diabetes (previously diagnosed at a government hospital or by a registered medical practitioner) was done using indirect ophthalmoscopy. A binary-logistic regression analysis was performed with ‘presence of DR’ as the dichotomous dependent variable and other independent covariates.

**Results:**

Crude prevalence of diabetes was 12.0% (n = 536), of which 344 were patients with ‘known’ diabetes. Mean age was 56.4 ± 10.9 years and 37.3% were males. Prevalence of any degree of DR was 27.4% (Males-30.5%, Females-25.6%; p = 0.41). In patients with DR, majority had NPDR (93.4%), while 5.3% had maculopathy. Patients with DR had a significantly longer duration of diabetes than those without. In the binary-logistic regression analysis in all adults duration of diabetes (OR:1.07), current smoking (OR:1.67) and peripheral neuropathy (OR:1.72) all were significantly associated with DR.

**Conclusions:**

Nearly 1/3rd of Sri Lankan adults with self-reported diabetes are having retinopathy. DR was associated with diabetes duration, cigarette smoking and peripheral neuropathy. However, further prospective follow up studies are required to establish causality for identified risk factors.

## Background

Diabetes mellitus is currently the commonest endocrine disorder, affecting nearly 6% of the world’s population [1]. According to recent estimates by the International Diabetes Federation, the number of patients with diabetes will increase by 55% to nearly 600 million by year 2035 [2]. The majority of the current 382 million people with diabetes are aged between 40 and 59, and 80% of them live in low- and middle-income countries like Sri Lanka [2]. Diabetes is accompanied by a host of micro- and macro-vascular complications, which further increases the disease burden by causing premature death and loss of productivity.

Diabetic Retinopathy (DR) is a common complication of diabetes mellitus. It is one of the leading cause of blindness worldwide, often affecting working-aged adults [3]. Globally the principal causes of visual impairment are uncorrected refractive errors (43%) and cataracts (33%), followed by glaucoma (2%), age related macular degeneration (1%) and diabetic retinopathy (1%) [4]. It is characterized by signs of retinal ischemia and/or increased retinal vascular permeability, with loss of vision due to neo-vascularization, hemorrhage, retinal detachment, macular edema and/or retinal capillary non-perfusion [3]. Patients with DR are 25 times more likely to become blind than those without diabetes [5]. Loss of productivity and reduced quality of life in patients with DR leads to an additional socioeconomic burden on the communities. Common risk factors for the development of DR include duration of diabetes, poor glycaemic control, elevated blood pressure, presence of diabetic nephropathy and dyslipidaemia [5,6].

South Asia, commonly known as the Indian subcontinent, is home to almost one-quarter of the world’s population. Sri Lanka is a middle income South Asian country with a population of about 21 million. Sri Lanka is experiencing a significant epidemic of diabetes with a rapid rise in prevalence over the last few decades [7]. Several recent studies from UK reported a higher prevalence of retinopathy in South Asians residing in UK than in the native Europeans [8,9]. However the current evidence regarding the epidemiology of DR amongst South Asians is conflicting, with most studies based on South Asians residing in European countries [8-11]. In the Sri Lankan context a study by Fernando et al. in 1993 on 1,003 patients with type 2 diabetes attending a clinic, showed that 31.3% had DR [12]. However, at present there are no large scale community based nationally representative studies from Sri Lanka evaluating prevalence and associations of DR among the native adult population. The present study aims to evaluate the prevalence of DR and its risk factors from a large cohort of community based South Asian adults with self-reported diabetes mellitus derived from a nationally representative sample from Sri Lanka.

## Methods

### Study population and sampling

A cross-sectional community based national study was conducted in seven of the nine provinces in Sri Lanka between August 2005 and September 2006 (Sri Lanka Diabetes and Cardiovascular Study – SLDCS). Detailed sampling is reported elsewhere [13]. In brief, five thousand non-institutionalized adults over 18 years of age were invited for the study using a multi-stage stratified cluster sampling technique. One hundred clusters (of 50 adults each) were selected to represent the seven provinces and clusters were divided amongst the provinces using a probability-proportional to-size (PPS) technique based on the total population of each province. In each province the clusters were selected from the ‘Village Office Units’ by a computer-generated random number list. Voter registration lists of the selected ‘Village Officer Units’ were used to randomly select the first household in each cluster and a uniform criterion was used to select the remaining 49 households. Ethical approval for the study was obtained from the Ethics Review Committee, Faculty of Medicine, University of Colombo, Sri Lanka. Informed written consent was obtained from all study participants and the study was conduct in accordance with Declaration of Helsinki.

### Data collection

Data collection was carried out by a trained team of medical undergraduates. An interviewer administered questionnaire was used to collect socio-demographic and anthropometric details including age, gender, area of residence, ethnicity, level of education, household monthly income (LKR – Sri Lankan Rupees), duration of diabetes, height, weight, waist circumference and hip circumference. Data on physical activity were collected using the short version of the International Physical Activity Questionnaire. Height was measured using Harpenden stadiometers (Chasmors Ltd, London, UK) to the nearest 0.1 cm, according to standard methods [14]. Body weight was measured using a SALTER 920 digital weighing scale (SALTER Ltd, Tonbridge, UK) to the nearest 0.1 kg. Body Mass Index (BMI) was calculated as weight in kilograms divided by height squared in meters (kg/m^2^). Waist circumference (WC) was measured midway between the iliac crest and the lower rib margin at the end of normal expiration and hip circumference was measured at the widest level over the greater trochanters using a plastic flexible tape to the nearest 0.1 cm. Waist to Hip Ratio (WHR) and Waist to Height Ratio (WHtR) were calculated as waist circumference divided by hip circumference and height respectively [15]. Seated blood pressure was measured after at least a 10-min rest with Omron IA2 digital blood pressure monitors (Omron Healthcare, Singapore). Fasting venous blood samples were obtained for glucose and lipid estimation from all participants, details of analysis have been previously described [13].

### Definitions

Patients were considered to be a ‘known’ diabetic subject if they had been previously diagnosed at a government hospital or by a registered medical practitioner. New cases (‘undiagnosed diabetes’) were diagnosed according to the American Diabetes Association (ADA) and World Health Organization (WHO) criteria diagnosis (Fasting blood glucose >7.0 mmol/l (126 mg/dl) but not reporting themselves as having diabetes”) [16,17]. Hypertension was defined as systolic blood pressure > 130 mmHg and/or diastolic blood pressure > 85 mmHg and/or being on anti-hypertensive treatment. Central obesity was classified as WC > 90 cm for males and >80 cm for females (Asian cut-offs). Obesity was defined as a BMI ≥ 27.5 kg/m^2^, based on WHO criteria for Asians [18]. Urban and rural sectors were defined according to the classification of the Sri Lankan government. Physical activity was classified in to three categories (‘Inactive’ , ‘Moderately active’ and ‘Highly active’) based on the total MET minutes/week derived from the IPAQ short version [19]. Peripheral neuropathy was assessed using the validated Toronto Clinical Scoring System (TCSS) (Score >5) [20].

### Ophthalmological examination

Ophthalmological evaluation of all patients with ‘known’ diabetes was done, while the remaining new cases (‘undiagnosed diabetes’) were not evaluated due to logistic reasons. All patients with ‘known’ diabetes had their pupils dilated with 1% tropicamide and was screened for DR by two Ophthalmologist by indirect ophthalmoscopy using slit lamp biomicroscopy, who equally shared the screening process and each patient was seen by only one ophthalmologist. Retinopathy was classified in to the following categories, according to the International Clinical DR Disease Severity Scale; normal, Non Proliferative DR (NPDR) (mild/moderate/severe), and proliferative DR, whilst presence/absence of macular edema (maculopathy) was also noted [21]. The presence of retinopathy in one eye was considered a diagnosis of DR and when asymmetrical DR was present the stage of retinopathy was based on the affected eye with the more severe grade of DR. Presence of Cataract was assessed clinically during slit-lamp examination using Lens Opacities Classification System (LOCS) III.

### Statistical analysis

Data were analysed using SPSS v14 (SPSS Inc., Chicago, IL, USA) statistical software package. The significance of the differences between proportions and means was tested using chi-square test and Student’s *t*-test/ANOVA respectively. Multiple groups were compared with post-hoc analysis using the fisher’s least significance difference (LSD) test. Unless otherwise stated age- and sex- standardized prevalence rates are presented throughout the manuscript. In all statistical analyses *p* < 0.05 was considered significant. Data were analysed using SPSS v14 (SPSS Inc., Chicago, IL, USA) statistical software package. Subjects were divided in to two groups based on the presence or absence of DR. A binary logistic regression analysis was performed in all patients with ‘presence of DR’ as the dichotomous dependent variable (0 = DR absent; 1 = DR present) and duration of diabetes (continuous), current smoking (binary), central obesity (binary) and peripheral neuropathy (binary) as the independent variables. The explanatory independent variables that were associated with the dependent variable in univariate analysis (p < 0.25) were selected to be included in the regression analysis. A similar binary logistic regression analysis with above dependant and independent variables was also performed separately for both males and females.

## Results

### Sample characteristics

Out of the 5000 invited subjects, 4485 participated in the study (response rate 89.7%). The crude prevalence of diabetes was 12.0% (n = 536), of which 344 (64.2%) were patients with ‘known’ diabetes. This report is based on 312 subjects with ‘known’ diabetes after excluding 33 subjects with incomplete data. Mean age (±SD) was 56.4 ± 10.9 years and 37.3% (n = 116) were males. The mean height (±SD), weight (±SD), BMI (±SD), waist circumference (±SD) and hip circumference (±SD) was 155.5 ± 9.2 cm, 57.7 ± 11.4 kg, 23.8 ± 3.8 kg/m^2^, 85.4 ± 10.6 cm and 92.9 ± 8.7 cm respectively. The prevalence of obesity (BMI ≥ 27.5 kg/m^2^) and central obesity (based on waist circumference) in the study population were 14.8% (n = 46) and 53.7% (n = 167) respectively. The prevalence of hypertension was 60.2% (n = 186), while metabolic syndrome was prevalent in 72.6% (n = 201). A comparison of socio-demographic, anthropometric and disease prevalence characteristics of patients with known diabetes and newly diagnosed diabetes is presented as (Additional file 1: Table S1), where only mean age and prevalence of hypertension were significantly different between the two groups.

### Prevalence, clinical and biochemical correlates of DR

The prevalence of any degree of DR in the study population was 27.4% (n = 76, 95% CI: 22.3 – 33.1). There was no significant gender difference observed in the prevalence of DR (Males 30.5%, Females 25.6%; p = 0.41). The prevalence of cataract was 10.9% (Males 9.5%, Females 11.7%; p = 0.52). In the patients with DR, majority had NPDR (93.4%, n = 71), while only one patient had PDR and 5.3% (n = 4) had maculopathy. Patients with DR had a significantly longer duration of diabetes than those without DR (Table 1). They also had a significantly lower waist circumference, waist to height ratio and diastolic blood pressure. However, other socio-demographic, anthropometric, clinical and biochemical parameters were not significantly different in those with and without DR (Table 1).

**Table 1:** Association of age, clinical and biochemical parameters with diabetic retinopathy

	**Diabetic retinopathy**		p value*
	**Absent**	**Present**	
	**Mean (±SD)**	**Mean (±SD)**	
Age (years)	55.3 (±10.2)	55.8 (±12.3)	0.73
Duration of diabetes (years)	5.5 (±4.7)	7.9 (±6.8)	0.001
Height (cm)	155.4 (±9.7)	156.4 (±8.5)	0.44
Weight (kg)	58.6 (±11.8)	57.0 (±10.5)	0.29
Body Mass Index (kg/m^2^)	24.2 (±4.0)	23.2 (±3.2)	0.06
Waist circumference (cm)	86.5 (±10.4)	83.3 (±9.2)	0.02
Hip circumference (cm)	93.7 (±8.9)	92.0 (±7.9)	0.167
Waist to hip ratio	0.91 (±0.06)	0.92 (±0.06)	0.06
Waist to height ratio	0.56 (±0.07)	0.53 (±0.05)	0.006
Systolic blood pressure (mmHg)	138.2 (±20.8)	136.0 (±22.6)	0.44
Diastolic blood pressure (mmHg)	81.1 (±11.1)	77.4 (±12.8)	0.02
Fasting blood glucose (mg/dl)	136.7 (±55.1)	145.1 (±56.8)	0.27
Total cholesterol (mg/dl)	215.9 (±43.8)	223.9 (±46.5)	0.18
LDL cholesterol (mg/dl)	137.2 (±40.6)	145.9 (±40.8)	0.11
HDL cholesterol (mg/dl)	45.7 (±8.6)	48.2 (±9.7)	0.06
Triglycerides (mg/dl)	164.8 (±105.9)	148.7 (±60.8)	0.21

*patients with and without diabetes retinopathy.

In all adults with diabetes the prevalence of DR was highest in urban residents (p-0.47), Sinhalese (p-0.52) and those educated up to primary school (Table 2). Current smokers (37.5%) had a significantly higher prevalence of DR than non-smokers (26.5%) (p-0.03). Similarly patients with diabetic peripheral neuropathy (30.4%) had a higher prevalence than those without neuropathy (20.2%) (p-0.006). However, DR was more prevalent in non-obese (p-0.03) and normotensive adults (p-0.06) (Table 2). No consistent relationship was observed between DR and levels of physical activity.

**Table 2:** Prevalence of retinopathy in association with socio-demographic, anthropometric, clinical and biochemical parameters

	**Prevalence of diabetic retinopathy % (95% CI)**		
	**All adults**	**Males**	**Females**
Area of residence			
Urban	27.9 (22.7 – 33.6)	36.8 (22.7 – 52.9)	19.6 (10.8 – 31.6)
Rural	26.6 (19.6 – 34.6)	26.9 (17.3 – 38.4)	28.4 (20.5 – 37.6)
Ethnicity			
Sinhalese	28.8 (24.1 – 33.9)	33.3 (23.9 – 43.9)	26.2 (19.5 – 33.8)
Tamil	20.0 (6.7 – 41.1)	14.3 (7.1 – 43.0)	25.0 (4.4 – 61.2)
Muslim	21.2 (11.3 – 34.7)	21.4 (5.8 – 47.9)	21.1 (7.1 – 43.3)
Level of education			
No formal education	25.0 (8.8 – 49.4)	50.0 (2.5 – 97.5)	20.0 (3.5 – 51.9)
Primary education	49.0 (37.4 – 60.7)^§^	50.0 (35.1 – 64.9)^§^	48.6 (32.5 – 64.9)^§^
Secondary education	21.6 (17.1 – 26.6)^§^	24.4 (15.8 – 34.8)^§^	19.8 (13.6 – 27.5)^§^
Tertiary education	41.7 (20.4 – 65.6)	45.5 (18.9 – 74.1)	0
Monthly income (LKR)			
< 7,000	28.6 (22.4 – 35.4)	40.6 (24.8 – 58.1)	24.8 (17.1 – 33.9)
7,000 – 24,999	28.7 (22.2 – 36.0)	29.6 (18.6 – 42.8)	27.9 (17.7 – 40.1)
25,000 – 49,999	17.6 (6.1 – 36.9)	18.2 (3.2 – 48.3)	16.7 (0.8 – 59.3)
≥ 50,000	0	0	0
Current smoking			
Non-smoker	26.5 (24.1 – 28.2)^§^	28.4 (23.4 – 33.9)^§^	25.6 (19.5 – 32.5)
Smoker	37.5 (32.5 – 42.5)^§^	37.5 (32.1 – 42.8)^§^	0
Physical activity			
Inactive	29.4 (19.8 – 40.7)	36.8 (17.8 – 59.7)	25.0 (12.3 – 42.0)
Moderately active	25.2 (19.0 – 32.3)	26.4 (15.9 – 39.4)	24.2 (14.8 – 35.9)
Active	28.8 (20.9 – 37.8)	33.3 (18.9 – 50.5)	26.9 (17.9 – 37.6)
Central Obesity*			
Absent	33.9 (26.0 – 42.4)^§^	36.1 (26.8 – 46.6)^§^	31.8 (26.5 – 35.8)^§^
Present	22.0 (15.9 – 29.2)^§^	22.7 (15.2 – 33.8)^§^	21.7 (17.6 – 26.3)^§^
Systolic hypertension (> 135 mmHg)			
Absent	29.1 (23.4 – 35.3)	33.9 (21.8 – 47.8)	26.5 (18.2 – 36.1)
Present	24.6 (18.4 – 31.7)	25.5 (14.6 – 39.4)	23.9 (14.3 – 35.9)
Diastolic hypertension (> 80 mmHg)			
Absent	28.4 (22.6 – 34.8)	31.0 (21.1 – 42.4)	27.1 (20.3 – 34.9)
Present	23.0 (13.7 – 34.7)	28.1 (14.5 – 45.4)	17.2 (6.6 – 34.2)
Hypertension^♯^			
Absent	33.9 (25.6 – 43.1)	37.5 (23.6 – 53.1)	31.9 (21.9 – 43.3)
Present	23.3 (17.3 – 30.2)	26.6 (16.8 – 38.3)	21.2 (14.0 – 30.1)
Peripheral neuropathy			
Absent	20.2 (15.7 – 24.7)^§^	20.0 (16.5 – 23.5)^§^	20.4 (11.2 – 32.6)
Present	30.4 (26.2 – 34.2)^§^	34.2 (24.1 – 45.7)^§^	28.0 (20.4 – 36.6)

^§^Values under each variable in a single column with the same superscript are significantly different from each other (p < 0.01); ^♯^systolic hypertension or diastolic hypertension or both; ^*^waist circumference > 90 cm for males and > 80 cm for females.

### Binary logistic regression analysis

The results of the binary logistic regression analysis in all adults using the dichotomous variable ‘presence of DR’ as the dependant factor and other independent variables are shown in Table 3. The overall model was statistically significant and the Cox & Snell R-Square and Nagelkerke R Square values were 0.254 and 0.363 respectively. The results indicate that the duration of diabetes (OR: 1.07), current smoking (OR: 1.67) and peripheral neuropathy (OR: 1.72) all were associated with significantly increased risk of having DR (Table 3). Presence of central obesity was associated with a reduced risk of having DR (OR: 0.55). However in the two genders only duration of diabetes in females and current smoking in men were significantly associated with an increase risk of DR (Table 3).

**Table 3:** Binary logistic regression analysis in all adults, males and females

	**Odds ratio (95% CI)**		
**Co-variants**	**All adults**	**Male**	**Female**
Duration of diabetes	1.07 (1.01 – 1.14)^#^	1.06 (0.97 – 1.17)	1.08 (1.00 – 1.16)^#^
Current smoking	1.67 (1.21 – 2.28)^#^	1.61 (1.20 – 2.31)^#^	NA
Peripheral neuropathy	1.72 (1.50 – 1.92)^*^	1.71 (0.78 – 3.75)	1.52 (0.67 – 3.66)
Central obesity	0.55 (0.31 – 0.97)^*^	0.52 (0.19 – 1.35)	0.59 (0.28 – 1.27)

^#^p < 0.05; ^*^p < 0.01; NA – Not associated.

## Discussion

To our knowledge, this is first study on the prevalence of DR in a large nationally representative community based sample of Sri Lankan adults. Our results show that the prevalence of DR in Sri Lanka is 27.4% with majority (93%) having NPDR, while cataract was prevalent in 10.9% of Sri Lankan adults with diabetes. Two previous clinic based studies from Sri Lanka in 1993 and 1998 showed a DR prevalence of 31.3% and 15.0% (newly diagnosed DM) respectively [12,22]. A more recent (year 2005–2006) clinic based study among adults with young-onset diabetes (onset < 40 years) demonstrated an overall DR prevalence of 18.1% [23]. A pooled analysis of 22,896 individuals with diabetes from 35 population-based studies (1980–2008) around the world showed an overall prevalence of 34.6% for any degree of DR [24]. In the same analysis a sub-group evaluation of South Asians demonstrated a pooled prevalence of 19.1% (n = 886). However, majority of South Asians in the above analysis were residing in countries outside of South Asia, with only 3 regional studies from India [24]. In the present study, more than 1/4th of the Sri Lankan patients with diabetes were found to have DR, a prevalence that is much higher than what is reported in recent population-based prevalence studies from individual South Asian countries (Table 4). Although higher prevalence values are reported in some clinic based studies, they are likely to be subjected to a significant selection bias. Differences in socioeconomic factors, including access to and the level of diabetes care, genetic susceptibility, racial differences in the effect of DR risk factors are some of the possible explanations for the observed disparity in the prevalence rates. Population-based studies incorporating host and environmental data are needed to further clarify the effect of race and ethnicity on DR prevalence [24].

**Table 4:** Prevalence of diabetic retinopathy in South Asian countries

**Country**	**Year of study**	**Study setting**	**Prevalence of DR % (number)**
Bangladesh [25]	2013^*^	Population based (regional)	21.6 (n = 20)
India [26]	2005^*^	Population based (regional)	17.6 (n = 302)
India [27]	2005 – 2006	Population based (regional)	12.2 (n = 342)
India [28]	2003 – 2006	Population based (regional)	18.0 (n = 255)
Nepal [29]	2005 – 2006	Hospital based	44.7 (n = 166)
Nepal [30]	2008^*^	Population based (regional)	19.3 (n = 11)
Nepal [31]	2009	Clinic based	20.3 (n = 26)
Pakistan [32]	2006 – 2008	Clinic based	27.4 (n = 460)
Pakistan [33]	2008 – 2009	Population based (regional)	12.0 (n = 183)
Pakistan [34]	2008 – 2010	Clinic based	25.5 (n = 51)

^*^Year of publication; DR – Diabetic Retinopathy.

Duration of diabetes, current smoking and presence of diabetes peripheral neuropathy all were significantly associated with the prevalence of DR in the present study population. Duration of diabetes is a well known risk factor for DR and most other complications of diabetes [24]. The relationship between smoking and DR is controversial, where several studies have shown no relationship between smoking and long-term incidence, progression of DR [35,36]. However these findings have been contradicted by other studies [37]. The failure to demonstrate a clear association between smoking and DR does not imply that persons with diabetes who smoke should not stop, as it is a well established vascular factor, associated with myocardial infarction and death from cardiovascular disease in patients with diabetes. Diabetic peripheral and autonomic neuropathy both are well known to be associated with DR, possible due to the fact that both are mediated by similar aetio-pathogenesis mechanisms [38,39].

Obesity is a well established risk factor for DR, because of its significant correlation to quality of metabolic control in patients with diabetes [40]. However, in the present cohort DR was more prevalent in the non-obese and patients with DR had a significantly lower waist circumference. It is possible that patients with DR in the present cohort had poor glycaemic control with resultant loss of weight and rapid progression of the retinopathy, resulting in DR being more prevalent in those without obesity. A similar result was observed in a small clinic based study of patients with Type-2 diabetes from Vanuatu islands, where patients with DR had a significantly lower BMI than those without [41]. Furthermore, although statistically not significant Fasting Blood Glucose values were higher in patients with DR than those without, however we lack HbA1c data to extrapolate on long term glycaemic control. Hypertension is another well known risk factor for DR [42]. However in the present cohort we did not observe a significant relationship between hypertension and prevalence of DR. In fact, DR was more prevalent in the normotensive diabetic population, although this difference was not statistically significant. In the present population with diabetes, nearly 2/3rd of the patients were hypertensive, making it likely that a high number of patients with and without DR were hypertensive irrespective of DR status.

There are several limitations to our study; the cross sectional design limits the inference of causality and can only demonstrates an association between DR and identified risk factors. Hence, prospective follow up studies among South Asian adults with uncomplicated diabetes is required to identify risk factors for DR during subsequent follow up. The lack of fundus photographs is also a limitation, since retinal photography is the gold standard for the diagnosis of DR; it is possible that we may have missed patients with early DR, hence underestimating the prevalence. However, in resource limited settings such as in Sri Lanka, ophthalmoscopy by experienced ophthalmologists also has been shown to have acceptable sensitivity [43]. In addition the grading given by the ophthalmologist was not validated by measuring intra/inter-observer concordance. In addition since the patients with newly diagnosed diabetes were not referred for eye examination, it could have been a source of selection bias and the prevalence of DR may have been overestimated. We were also unable to evaluate data on treatment, including insulin therapy, glycaemic control and their associations with DR. Furthermore, we did not have complete data for the prevalence of nephropathy to evaluate its association with DR.

## Conclusions

Our results demonstrate that nearly 1/3rd of Sri Lankan adults with self-reported diabetes are having retinopathy. Diabetic retinopathy was associated with duration of diabetes, cigarette smoking and peripheral neuropathy. However, further prospective follow up studies among South Asian adults are required to establish causality for the risk factors identified.

## Competing interests

The author(s) declare that they have no competing interests.

## Authors’ contributions

PK made substantial contribution to conception and study design and data collection. PK, PR, and RJ were involved in refining the study design, statistical analysis and drafting the manuscript. PR and RJ critically revised the manuscript. All authors read and approved the final manuscript.

## Pre-publication history

The pre-publication history for this paper can be accessed here:

http://www.biomedcentral.com/1471-2415/14/100/prepub

## Supplementary Material

Additional file 1: Table S1 Comparison of socio-demographic, anthropometric and disease prevalence characteristics of patients with known diabetes and newly diagnosed diabetes. Click here for file
