# Supplementary material for: Prevalence of retinopathy among adults with self-reported diabetes mellitus: the Sri Lanka diabetes and Cardiovascular Study
Source: BMC Ophthalmol. 2014 Aug 20;14:100. doi: 10.1186/1471-2415-14-100 (PMC4141126; doi:10.1186/1471-2415-14-100)
Supplement: Additional file 1: Table S1 — Comparison of socio-demographic, anthropometric and disease prevalence characteristics of patients with known diabetes and newly diagnosed diabetes. [file 1471-2415-14-100-S1.doc]

|  | Known diabetes | Newly diagnosed diabetes | P value |
| --- | --- | --- | --- |
| Mean age (years) | 56.4±10.9 | 51.5±13.5 | <0.001 |
| Gender  Males (%)  Females (%) | 37.3  62.7 | 38.0  62.0 | 0.75 |
| Area of Residence  Urban (%)  Rural (%) | 36.0  64.0 | 33.2  66.8 | 0.94 |
| Mean height (cm) | 155.5±9.2 | 155.0±8.5 | 0.78 |
| Mean weight (kg) | 57.7±11.4 | 57.5±12.8 | 0.99 |
| Mean Body Mass Index (kg/m^2^) | 23.8±3.8 | 23.8±4.3 | 1.00 |
| Mean waist circumference (cm) | 85.4±10.6 | 83.8±11.1 | 0.10 |
| Mean hip circumference (cm) | 92.9±8.7 | 92.7±9.4 | 0.85 |
| Obesity prevalence (%) | 14.8 | 15.4 | 0.14 |
| Hypertension prevalence (%) | 60.2 | 46.9 | <0.001 |
| Metabolic syndrome prevalence (%) | 72.6 | 72.9 | 0.82 |

**Additional file 1**

**Table S1:** Comparison of socio-demographic, anthropometric and disease prevalence characteristics of patients with known diabetes and newly diagnosed diabetes
